# Author Correction: Isolated detection of elastic waves driven by the momentum of light

**DOI:** 10.1038/s41467-019-11969-1

**Published:** 2019-08-28

**Authors:** Tomaž Požar, Jernej Laloš, Aleš Babnik, Rok Petkovšek, Max Bethune-Waddell, Kenneth J. Chau, Gustavo V. B. Lukasievicz, Nelson G. C. Astrath

**Affiliations:** 1 Faculty of Mechanical Engineering, University of Ljubljana, Ljubljana, 1000 Slovenia; 2 School of Engineering, The University of British Columbia, Kelowna, BC V1V-1V7 Canada; 3 Department of Physics, Universidade Tecnológica Federal do Paraná, Medianeira, Paraná, 85884-000 Brazil; 4 Department of Physics, Universidade Estadual de Maringá, Maringá, Paraná, 87020-900 Brazil

**Keywords:** Optical physics, Photoacoustics

Correction to: *Nature Communications* 10.1038/s41467-018-05706-3, published online 21 August 2018.

The original version of this Article contained an error in the *x*-axis labels of Fig. 3a, b, which incorrectly read ‘*z* (mm)’. The correct version states ‘*z* (nm)’. This has been corrected in both the PDF and HTML versions of the Article.

