# Supplementary material for: Isolated detection of elastic waves driven by the momentum of light
Source: Nat Commun. 2018 Aug 21;9:3340. doi: 10.1038/s41467-018-05706-3 (PMC6105914; doi:10.1038/s41467-018-05706-3)
Supplement: Supplementary file 1 — Description of Additional Supplementary Files [file 41467_2018_5706_MOESM1_ESM.pdf]

Description of Additional Supplementary Files

File Name: Supplementary Movie 1

Description: Linear momentum deposition from a light pulse to elastic waves with their subsequent propagation and detection
